# Mineral Composition of Cereal and Cereal-Free Dry Dog Foods versus Nutritional Guidelines

**DOI:** 10.3390/molecules25215173

**Published:** 2020-11-06

**Authors:** Katarzyna Kazimierska, Wioletta Biel, Robert Witkowicz

**Affiliations:** 1 Department of Monogastric Animal Sciences, Division of Animal Nutrition and Food, West Pomeranian University of Technology in Szczecin, 29 Klemensa Janickiego, 71270 Szczecin, Poland; wioletta.biel@zut.edu.pl; 2 Department of Agroecology and Crop Production, University of Agriculture in Krakow, 21 Mickiewicza, 31120 Krakow, Poland; robert.witkowicz@urk.edu.pl

**Keywords:** comparison analysis, element ratios, extruded dog food, ingredients, macroelements, microelements, mineral profile, nutritional value

## Abstract

The aims of the present work are to estimate the nutritional value and to evaluate and compare the levels of macroelements (Ca, P, K, Na, Mg), microelements (Fe, Zn, Mn, Cu), heavy metals (Co, Cd, Pb, Mo, Cr, Ni), and their ratios in extruded complete foods for adult dogs, their compatibility with nutritional guidelines, as well as food profile similarity. Basic composition was determined according to Association of Official Analytical Chemists (AOAC). Analyses for elements were performed using an atomic absorption spectrometer. All the evaluated dry dog foods met the minimum recommended levels for protein and fat. Eighteen tested dog foods (60%) did not meet at least one recommendation of nutritional guidelines. Four dog foods exceeded the legal limit of Fe and five foods exceeded the legal limit of Zn; in one of them, Zn level was almost twice higher. Dog foods with insect protein exceeded the legal limit for Mn content. Eight dog foods had an inappropriate Ca:P ratio. Heavy metals were below detection limit in all analyzed dog foods. The results seem to show the need for regular feed analyses of the elemental composition in raw materials before introducing supplementation and for the monitoring of the mineral composition of finished pet food.

## 1. Introduction

Companion animals’ population is increasing worldwide. According to the American Pet Products Association (APPA) [1], in 2018 67% of U.S. households, i.e., an estimated 84.9 million homes, owned at least one pet. In Europe this figure is estimated at 38% of all households (85 million homes) [2]. An increasing share of these impressive numbers of animal owners are becoming more aware of the nutritional needs of their animals. The constant demand from owners for better quality products means that the pet food sector is becoming particularly aware of providing nutritious food for animal health and welfare. The European Pet Food Industry Federation (FEDIAF) [3] established nutritional guidelines, based on the recommendations of the National Research Council [4] and scientific research. FEDIAF also works closely with the Association of American Feed Control Officials (AAFCO).

Other than the major nutrient requirements, companion animals require certain amounts of elements that must be provided in their food for a healthy, balanced diet. Calcium, phosphorus, and magnesium are associated with the development and maintenance of bones and teeth, as well as being necessary for the proper functioning of the muscular and nervous system of dogs [5]. Potassium and sodium are responsible for maintaining the electrolyte balance, regulation of body fluids, and transport of nutrients in the blood [6]. Nutritional guidelines provide minimum and, in some cases, maximum recommended levels for the elements that are essential in a dog’s diet (Ca, P, K, Na, Cl, Mg, Cu, I, Fe, Mn, Se, Zn). Their quantities in dog food should be monitored and are verified in scientific research [7,8,9,10,11,12,13], as it is important for animal heath safety [14,15,16].

While major macroelements and trace elements in pet foods are regulated by the US NRC [4], the AAFCO [17] and in Europe by FEDIAF [3], there are no standards set by regulatory agencies for monitoring ultratrace minerals such as chromium, nickel, molybdenum, cobalt, cadmium, lead, silica, and aluminum. Animal feeds and feed materials may be contaminated with undesirable substances that may come from polluted environments and/or the production process. There are even report documenting the presence of heavy metals in pet food [18,19,20]. Higher levels than normal probably originate from ingredients that have been exposed to environmental pollution and bioaccumulation over time [21]. Contemporary pet food formulations use various foods as their main ingredients, including poultry, fish, ruminants and monogastric animal meat, plant seeds (legumes, cereals), and plant biomass. The raw materials used for production, types of agrotechnical production of the plants, and the place of their geographical cultivation affect the level of minerals in the final product.

It is worth noting that there is a substantial scientific interest in the topic of grain-free trend in pet food sector [22,23]. For many years the phrase “grain-free” has been widely used in pet food market. However, it seems that “grain-free” is a marketing term rather than scientific definition. Plants considered “grain crops” are those producing small, hard dry seed or fruit consumed by man or his domesticated animals as a foodstuff, or processed for food or industrial purposes [24]. On the basis of the Encyclopedia of Grain Science [24] grains include, among others, green beans, sugar peas, lupins, amaranth, and linseeds. Therefore, when it comes to the presence or absence of cereals in the composition, more scientifically correct phrase seem to be “cereal-free”.

Most domestic dogs in industrialized countries are fed extruded dog food [25]. The information provided on the dog food label should be helpful for the owner and include detailed information concerning not only the ingredients list, but also the nutritional value (incl. nutrient levels and mineral content) and feed additives used. Since pets rely on commercial pet foods for daily energy and nutritional requirements, long-term consumption of the foods may lead to an excess or deficiency of minerals depending on the product.

The aims of the present work are to estimate the nutritional value and to evaluate and compare mineral profiles, heavy metal concentrations, mineral ratios, and profile similarity in randomly selected extruded maintenance commercial complete food for adult dogs with a particular emphasis on the division into cereal and cereal-free foods, and also to establish if the mineral levels meet the recommended requirements.

## 2. Results

### 2.1. Basic Composition

All tested dog foods met the recommended minimum levels for crude protein and fat according to FEDIAF [3], which are 18 g and 5.5 g/100 g of dry matter (DM) of food, respectively (Table 1). The analyzed dog foods differed in the content of protein and ether extract (range 21.4–38.97 and 6.31–21.39 g/100 g DM, respectively). Crude fiber content varied in analyzed food and ranged from 2.9 to 15.14 g/100 g DM. Crude ash ranged from 4.8 to 9.92 g/100 g DM. Carbohydrate content (defined as nitrogen free extract, NFE) was significantly different between the tested dog foods and ranged from 17.74 to 53.17 g/100 g DM. The metabolizable energy (ME) content in the dog foods also varied and ranged from 347 to 415 kcal ME/100 g DM. Differences in protein, fat, fiber, ash, and NFE content were seen between those with a presence or an absence of cereal in the composition. Cereal-free dog foods had more crude protein (+19%), fat (+30%), fiber (+33%), and ash (+11%) than foods with cereals. In turn, cereal foods had more NFE (+32%) than cereal-free dog foods.

### 2.2. Macroelement Levels

**Figure 1:**
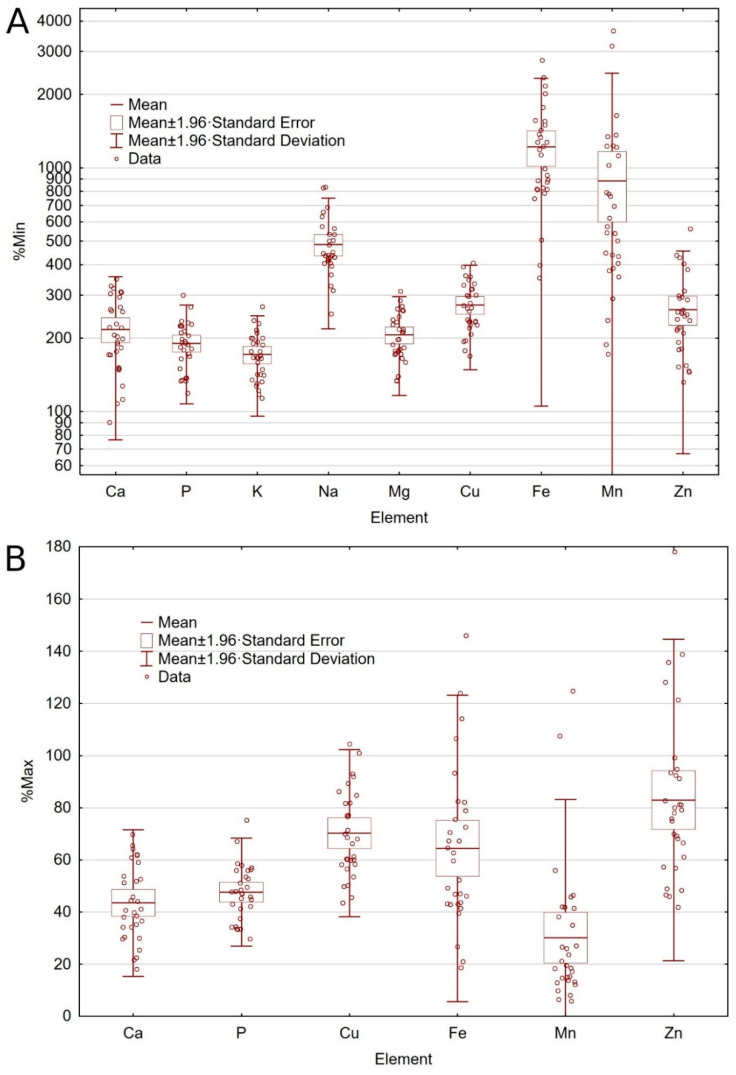
Amount of minerals in relation to the minimum recommended level (**A**) and maximum nutritional and maximum legal limit (**B**) contents according to European Pet Food Industry Federation (FEDIAF) [3].

One out of thirty analyzed dog foods did not meet the minimum recommended level (MRL) for Ca (Table 2, Figure 1A). Sixteen tested foods (53%) had more than twice the MRL of Ca and eleven dog foods (37%) had more than twice the MRL of P (Table 2, Figure 1A), although all values were within the established maximum nutritional limit (MNL) (Table 2, Figure 1B). All dog foods met the MRL for K, Na, and Mg. No MNLs have been established by FEDIAF [3] for the K, Na, and Mg. However, as reported by FEDIAF [3], Na levels up to 1.5% of DM are safe for healthy dogs and higher levels may still be safe, although no scientific data is available. The MRL was exceeded more than three times for Na in twenty-nine tested foods (97%, Table 2, Figure 1A), though no dog food exceeded the amount of 1.5 g/100 g DM. For Mg, the MRL exceeded by more than double in fourteen tested foods (47%, Table 2, Figure 1A). It is worth noting that the recommended values given in Table 1, Table 2 and Table 3 are minimum recommended allowances for commercial pet food, not minimum requirements or optimal intake levels. In turn, the nutritional maximum is the highest level that should not cause any harmful effects and the maximum legal limit (MLL) is mandatory and the highest legally permitted amount of the nutrient. Among the tested foods significant differences in the presence or absence of cereals in the composition were found. Significantly more P, K, and Na were found in cereal-free foods. No statistically significant differences were found in the content of Ca and Mg (Table 2).

### 2.3. Trace Element Levels

All dog foods contained higher amounts of trace elements than the MRL established by the nutritional guidelines (Table 3). Two out of thirty foods exceeded the MLL of Cu. Sixteen tested foods (53%) contained ten times more Fe than the MRL, of which four foods exceeded the MLL for Fe (Table 3, Figure 1A). Two tested dog foods exceeded the MRL of Mn by more than thirty times and exceeded the MLL (Table 3, Figure 1B). The Mn excess was in dog foods with insects as a main protein source (8% and 25% above MLL). Moreover, the Mn content in ten tested foods (33%) exceeded the MRL over ten times (Table 3, Figure 1A). Five dog foods exceeded the MLL of Zn. Values exceeded the recommended level by between 21% and 78% (Table 3, Figure 1B).

Significant differences were found between foods with a presence or absence of cereals in the composition. Significantly more Fe, Zn, Mn, and Cu were found in cereal-free foods (Table 3). Heavy metals (Co, Cd, Pb, Mo, Cr, Ni) levels were below limit of detection in the tested dog foods.

### 2.4. Trace Elements Additives

In the tested dog foods, either none, two or four feed nutritional additives were declared by the producers on the label (Table 4, Table S1). The trace elements labeled as additives contributed to 3.11 –85.82% of the total determined, the remaining being supplied by the raw materials (Table 4). However, the study showed that in some tested dog foods the amount of declared minerals on the label exceeded the results obtained in our research. This mainly concerned Mn (>100% from additives, *n* = 4), Zn (*n* = 4) and Cu (*n* = 1). On the other hand, in several products (*n* = 9) the manufacturer did not declare the addition of Fe and Mn on the label, consequently 100% of the results obtained in present study were obtained through raw materials. Five dog foods have declared the content of these four trace elements under the heading “analytical constituents” (reflecting total amount of the nutrient analyzed in the finished product, Table S1).

### 2.5. Comparative Analysis

In order to generalize the differentiation of ash composition of the tested foods, a comparison was made of the multivariate mineral composition profiles (Table 5). Out of 435 presented Cohen coefficients, as many as 195 (44.83%) were ±0.30 (no similarity), which means that the remaining 240 coefficients described dissimilarities or similarities of the analyzed mineral profiles of dog foods (34 (7.82%) and 206 (47.35%), respectively). This percentage distribution of the similarity index indicates a clear distinctiveness of several foods, which are characterized by negative similarity coefficients (negative similarity (dissimilarity)—red) and additionally by a lack of similarity (white). The first one in numerical order is food no. 1, generally characterized by the lack of similarity with 21 other foods, dissimilarity with 3 and similarity with 5 other feeds. The next is food no. 8, with its mineral composition profile being consistent only with food no. 30 (*r_c_* = 0.94) and food no. 9 (*r_c_* = 0.55). In relation to other foods, there was either a lack of similarity (18×) or dissimilarity (9×). The next two foods with a significant dissimilarity of mineral profiles compared to the other feeds are food nos. 11 and 12. Just like food no. 8 mentioned above, these foods have potatoes in their composition and at the same time belong to the group of cereal-free foods. Food no. 12 has a particularly different profile as it has 13 negative similarity coefficients, both in relation to foods with and without cereals, and 12 dissimilarity coefficients.

**Figure 2:**
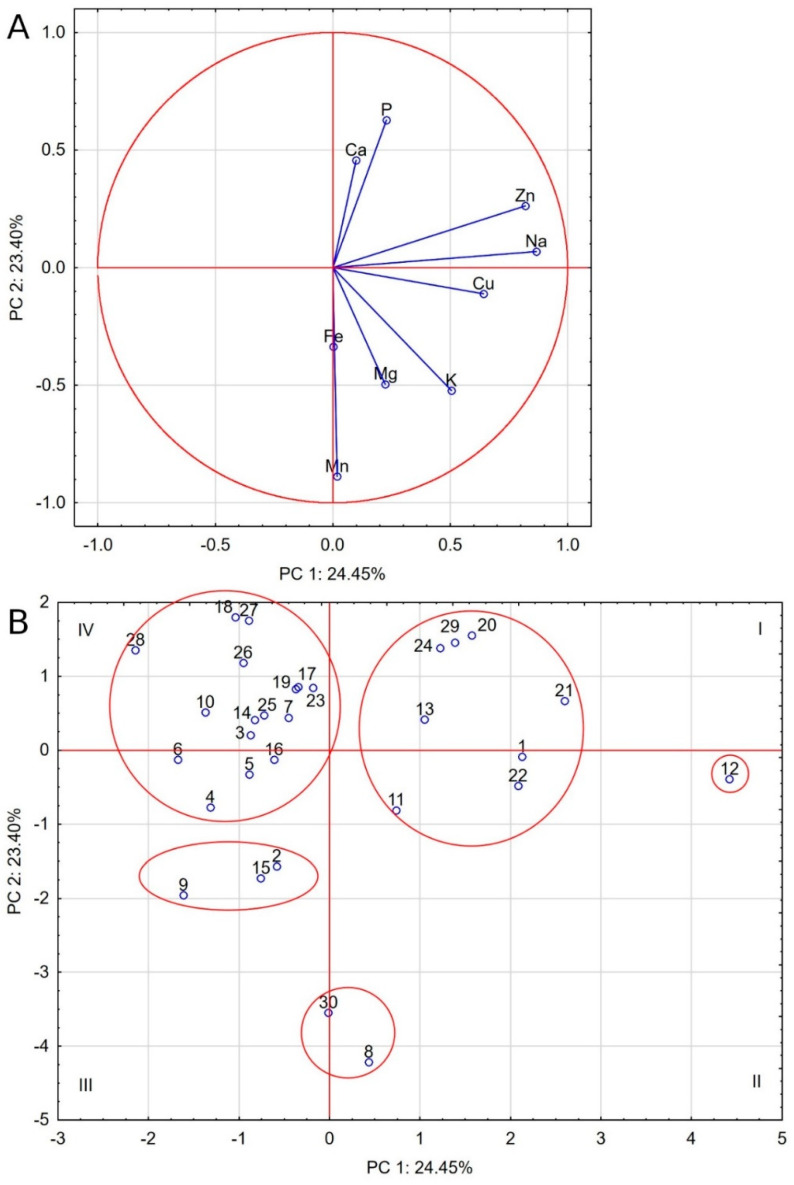
Biplot based on first two principal component axes for mineral composition of dog foods (**A**) and distribution of 30 commercial dog foods based on the first two components obtained from principal component analysis (**B**).

Similar observations come from the PCA analysis, but it should be stressed that its first two components only accounted for 47.85% of the total variance. Nevertheless, five groups of food were distinguished, as shown by the ellipses in Figure 2. The least populated group comprised of food nos. 8 and 30, with a high Cohen similarity index and showing elevated Mn levels strongly correlated with Fe and Mg levels. The second group comprises seven foods (nos. 1, 11, 13, 20, 22, 24, and 29). They share high levels of Na, Cu, Zn, P, and Ca and moderate levels of K and Mg. One absolutely distinct food is food no. 12, with even higher levels of Zn, Na, and Cu, negatively correlated with Mn, Fe, Ca, and P. Another group includes food nos. 2, 9, and 15, despite the fact that food no. 2 is the only cereal-free food in this group. The last group is made up of foods located mainly in the fourth quarter, with low levels of Na, Cu, K, Mg, and Mn. They are the group of dog foods with the most differentiated composition, as they include both cereal and cereal-free foods.

### 2.6. Relations between Elements

**Figure 3:**
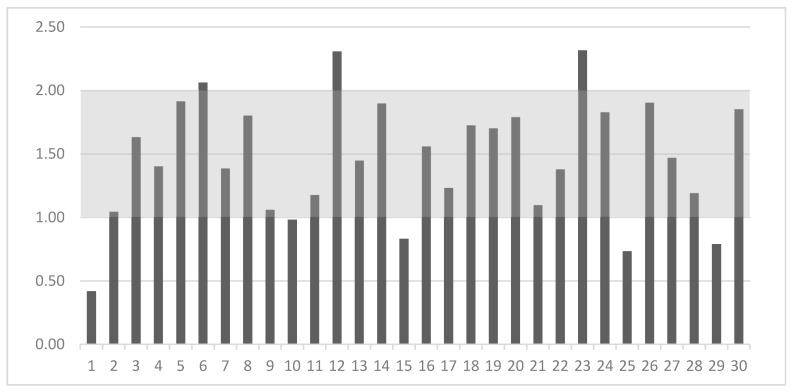
Calcium to phosphorus ratio (on *Y*-axis) in the 30 analyzed dry dog foods (on *X*-axis) and their comparison with nutritional guidelines [3] (gray area).

The nutritional guidelines have only established MRL and MNL for the Ca:P ratio, which is shown as the gray area in Figure 3. In our study, eight out of thirty dog foods (27%) had inappropriate Ca:P content. Three of them had a ratio above the MNL of 2:1. Five dog foods had a ratio in the range of 0.42–0.98:1, which seems insufficient in comparison to the MRL 1:1 recommended by FEDIAF [3]. One of them had a ratio more than two times lower than the MRL (Figure 3).

Although calculations are preliminary and more research is needed to establish appropriate mineral ratios, in the tested dog foods some incompatibilities with the determined ratios of individual mineral components were found (Table 6). Most derogations were related to the P:Zn ratio, in which twenty-five dog foods showed a lower ratio than the minimum ratio calculated based on the minimum recommended levels (ranging from 30% to 98% of the minimum ratio). Also the K:Mg, K:Ca, and Ca:Mg ratios showed large deviations from the calculated ratios (twenty-two, twenty and thirteen analyzed foods below the minimum calculated ratio, respectively). All tested dog foods meet the calculated minimum Na:K ratio. In the case of trace element ratios, three dog foods had an Fe:Mn ratio below the calculated minimum ratio (ranging from 50% to 59% below) and another three dog foods in the Mn:Zn ratio (ranging from 12% to 50% below).

## 3. Discussion

In Europe, the most commonly used recommendations for the chemical composition of pet foods and their recommended minimum levels and nutritional or legal maximum limits come from the FEDIAF [3]. Minimum nutrient requirements are defined as the lowest intake that will support normal function, such as maximal growth rates or prevention of deficiency symptoms.

### 3.1. Basic Composition

Of key importance is the source of protein, with its quality being decisive for the nutritive value of dog food [26]. An interesting and relatively new source of protein in dog food are insects, mainly from the order of *Diptera* and *Coleoptera*. Insects are considered as a sustainable protein source for future pet foods. Bosch et al. [27] concluded that the protein quality, determined based on the amino acid profile and digestibility of *Hermetia illucens*, *Musca domestica,* and *Tenebrio molitor*, was high and the undigested insect fractions were at least partly fermented by the dog’s fecal microbiota. Therefore, insect protein could be potentially included in pet food as health-promoting ingredients. In our study, dog foods with *Hermetia illucens* larvae as the main animal protein source had an average protein level of 22.36 g/100 g DM.

Commercial dog foods contain lipids from plant oils (mainly sunflower), and/or animals fat (e.g., chicken or fish), or often a mixture of both. Which fat sources a manufacturer will include in a dog food depends on several factors, such as essential fatty acid (EFA) content, effect on palatability, susceptibility to oxidation, and market price [28]. In the present work all tested dog foods had animal fat as their main fat source (see Table 5). In our study the average fat content of the tested dog foods was 13.24 g/100 g DM, which is confirmed by research conducted on twelve brands of dog food sold in Norway (average 13.5 g/100 g DM) [28].

Carbohydrates are a group of compounds and include digestible sugars (monosaccharides and disaccharides), starch (polysaccharides), and non-digestible fibers (oligo and polysaccharides). Digestible carbohydrates are a direct source of glucose, an important source of energy for the body, and the main source for some tissues like the brain and red blood cells. Dietary fiber is physiologically important for pets. Certain fermentable fibers, defined as prebiotic fibers, promote the development of a beneficial gut microflora, which can help reduce the colonization of harmful bacteria [29]. Depending on the plant raw material used for production, the composition of the fiber fraction, as well as concentration of volatile compounds [30] in the final product is different. Comparing cereal and cereal-free foods for dogs, Pezzali and Aldrich [31] concluded that dietary fiber digestibility was 31.9% greater for dogs fed a diet that did not include cereals. Moreover, dogs preferred cereal-free food (with potato, peas and tapioca) over food with cereals (spelt, millet, and sorghum) in the palatability assessment [31]. In turn, in another work, the aim of which was determination of volatile compounds in cereal and cereal-free dry dog foods, showed that cereal-free products are less aromatic [32]. Therefore, it is worth paying attention to the source of carbohydrates when choosing dog food. In our study, tested foods without cereals as the main source of plant carbohydrates contained: potato (*n* = 7), sweet potato (*n* = 5), and peas (*n* = 5) and had less NFE (average 6.71 g/100 g DM) than foods with cereals (average 7.45 g/100 g DM). Subsequently, the mean of crude ash content of all tested dog foods (7.13 g/100 g DM) was close to the results of a study of 20 commercial dry foods for adult dogs by Pereira et al. [11] (7.98 g/100 g DM).

### 3.2. Macroelements

Both the deficiency [33] and the excess [34,35] of Ca can lead to disorders in skeletal development. In a study conducted by Gagné et al. [36], in four dry dog foods (9%) Ca concentrations exceeded recommended limits. Pereira et al. [11] in the analyzed dry dog foods, also ascertained higher amounts of Ca than the maximum recommended levels, in both adult dog and puppy foods (values over 39% and 54% above the maximum limit, respectively). More deviations from the guidelines were found in a study of home-prepared diets for dogs, where sixty-two of the analyzed diets (83%) had Ca levels below the minimum recommended [37]. Although in our study most analyzed dog foods were in compliance with FEDIAF guideline, one food out of the thirty (3%) did not meet the MRL. The above-mentioned studies emphasize the need to feed a properly composed diet for specific life stages, especially for puppies of large and giant breeds.

After Ca, P is the second most prevalent mineral in mammals, including dogs and cats. It has been shown that P deficiency during the puppy period leads to slower growth and disturbances in the development of the musculoskeletal system [38]. In studies conducted on forty-five dry foods formulated for maintaining healthy dogs in the United States, three of them exceeded the recommended maximum concentration [36]. In turn, home-prepared diets once more showed a greater deviation from the FEDIAF guidelines, with 53% of the diets tested (40 diets out of 75) having P levels below the minimum amount recommended [37]. In this study all tested foods were in compliance with FEDIAF [3] recommendation, both in terms of minimum and maximum recommended levels. It is worth remembering that high levels of P may be due to the composition of mainly fresh meats [39], since animal protein contains more P than plant protein. In our study, analyzed dog foods with the highest P content (ranging from 0.938 to 1.204 g/100 g DM) were also characterized by a high protein content, above 30% of DM (ranging from 34.14 to 38.97 g/100 g DM). In particular, results for dog food nos. 21 and 29 may suggest that the ingredients are mostly animal-based (different species of fish).

In veterinary medicine, hypermagnesemia is much less clinically significant than a Mg deficit. However, since Mg is mainly excreted in the urine, it is recommended to avoid excessive Mg supplementation, especially in dogs with renal insufficiency [40]. In the present work, all analyzed foods had suitable Mg levels and the average content of Mg in the analyzed foods was similar in cereal and cereal-free foods (0.144 and 0.145 g/100 g DM, respectively).

Although a safe upper limit for the K content in dog food has not been established by nutritional guidelines, it should be taken into account that an increase in blood K levels (hyperkalemia) can be life-threatening because of the risk of cardiac arrhythmias. Therefore, excessive K supplementation should be avoided, especially in dogs with heart disease and chronic kidney disease. Appropriately formulated, potassium-reduced diets are an effective alternative to treat hyperkalemia [41]. In our study, all analyzed foods met the MRL for K, of which thirteen dog foods had 1.5 times more K than the MRL (Table 2, Figure 1A).

The situation was different with Na, where all analyzed foods had from about 2.5 to 8 times the amount specified in the MRL (Table 2, Figure 1A). Despite the lack of established maximum recommended values, FEDIAF [3] provides, that scientific data show that Na levels up to 1.5% DM are safe for healthy dogs. Healthy dogs appear to be able to adapt to varying amounts of Na in their diet through the renin-angiotensin-aldosterone mechanism [42]. Nonetheless, there is a lack of information on the effects of an excess or deficiency of Na in a dogs nutrition. Still, the effect of high Na content in pet foods is controversial, especially for pets that have systemic arterial hypertension, where it becomes advisable to avoid excessive consumption of this mineral [43]. Na excess, in addition to heart disease, chronic kidney disease, damage to the gastric mucosa and stomach cancer, may also impair Ca homeostasis by increasing risk factors for Ca oxalate formation [44]. Such high amounts of Na can therefore pose a health risk to dogs, and excessive amounts of Na in dog food have previously been reported [39]. Further studies are needed to investigate what concentrations are safe for dogs.

It is worth noting that the mandatory declaration of analytical constituents is related to the type of feed and/or the target animal species [45]. For complete feed for pet animals like cats and dogs, analytical constituents of minerals may be labelled voluntarily. Although manufacturers are not required to provide the content of individual macro- and microelements under the heading “Analytical constituents,” many products contain such information, especially calcium and phosphorus content (Table S2).

### 3.3. Trace Elements

While Cu plays an important role in a variety of biological processes, including mitochondrial respiration, antioxidant defense, neurotransmitter synthesis, connective tissue formation, pigmentation, and Fe metabolism [46], when consumed in excessive amounts it can also cause acute or chronic Cu poisoning. Excess Cu accumulation in the dog’s liver may cause hepatitis and cirrhosis [47]. Acute poisoning is usually seen after the accidental administration of excessive amounts of soluble Cu salts, which may be present in mineral mixes or improperly formulated rations in dog’s diets. Commercial dog food can also be a source of excessive amounts of Cu. For example, half of the dog foods tested by Pereira et al. [11] were overloaded with Cu by more than thirty times the amount recommended by the NRC [4]. In our study, two out of thirty dog foods had amounts of Cu above the MLL established by FEDIAF [3] (1% and 5% above limit), though a number of tested dog foods had over 2.5 times more Cu than the MRL (Table 3, Figure 1A). In most tested foods, Cu mainly came from the raw materials used in production, not from any nutritional additives (Table 4). However, in foods with excessive Cu content, the concentration of this element was enriched with nutritional additives, which possibly led to its excess.

Iron is necessary for the synthesis of oxygen-transporting proteins, particularly hemoglobin and myoglobin, and for the formation of heme enzymes and other Fe-containing enzymes involved in electron transfer and oxidation-reductions [48,49]. Deficiency results when either dietary intake does not meet the body’s requirement or when there is chronic external (non-resorptive) blood loss, leading to anemia [15]. Iron excess may be even more harmful and can result in serious toxicity. High oral doses of Fe can overcome the normal rate-limited absorption by saturating iron-binding proteins. Abnormalities due to Fe overdose include dehydration, hypovolaemia, anemia, signs of liver necrosis and liver failure [50]. There is no physiological pathway for excretion of excess iron, therefore, regulation of the amount of iron absorbed from the diet plays a key role in maintaining the iron balance [51,52]. In this study, all analyzed foods were generally rich in Fe, as dog food with the lowest Fe content exceeded the MRL 3.5 times anyway, and the food with the highest Fe content almost 30 times (Table 3). Sixteen analyzed foods (53%) contained ten times more Fe than the MRL (Table 3, Figure 1A), of which four foods exceeded the MLL (Figure 1B). In turn, in the study obtained by Davies et al. [53] all the tested dry foods met the minimum and maximum levels for the quantity of Fe (in compliance with FEDIAF, 2013 [54]). What is important, the evaluation of the results of this study was influenced by the update of the nutritional guidelines. Comparing the obtained Fe content in present study with the FEDIAF guidelines from 2019 [55], when MLL for Fe was 142 mg/100 g DM, all the tested foods met the recommended levels for this element. However, the update decreased the MLL for Fe by more than half, since current limit for Fe is 68.18 mg/100 g DM [3], and for this reason, four tested foods exceeded the recent MLL. Furthermore, the amount of Fe in the analyzed foods is primarily derived from the raw materials used, not from nutritional additives (Table 4). Dog foods contained from 3.11% to 38.39% of Fe from nutritional additives; moreover, nine of the analyzed foods did not declare any Fe added at all on the label, which means that 100% of the Fe came from the raw materials that were used. In turn, in dog foods with exceeded Fe content, the concentration of this element was additionally enriched with nutritional additives. Such large amounts of Fe may indicate a high proportion of Fe-rich offal [56], but the ingredients list of the food is not precise enough to be able to determine this.

There is a fairly wide acceptable range for recommended Mn levels [3]. However, in ten of the tested dog foods (33%), the Mn content exceeded the MRL by more than 10 times (Table 3, Figure 1A) of which two dog foods exceeded the MRL more than 30 times, exceeding the specified MLL at the same time (Figure 1B, Figure 2). Analyzed foods with an excessive amount of Mn were dog foods based on insect protein. Also, the third dog food with insects had a high amount of Mn, differing in the amount from the other tested foods (Table 3). Interestingly, in these dog foods, the amount of Mn does not come from nutritional supplements at all or comes from them in a small amount (14% from nutritional additives, Table 4). This may indicate a large amount of Mn from raw insect products. Many insect species have high concentrations of Mn in the body because, among others, the deposition of Zn and Mn in the larvae’s mandibles increases the larva’s ability to penetrate seeds [57,58]. Data also suggest that Mn exposure affects general locomotion, as well as innate behaviors associated with feeding drive and food choices, although the direct impact of Mn on insect behavior has been studied in just a few species [59]. In turn, in the species *Hermetia illucens*, i.e., the species whose larvae were used in the production of the tested dog food (see Table 5), it was shown that the relatively high total content of Mn in larvae (Mn^2+^ content 433.0 ± 14 mg/kg biomass of the *Hermetia illucens*) points to an important role of this metal in its ontogeny [60]. Therefore, it can be assumed that dog food with insects as the main source of protein will contain high levels of Mn, which was shown by our research. Chronic Mn exposure leads to its accumulation in the basal ganglia, pallidum, and striatum regions of the mammalian brain, with subsequent neurotoxic effects on the dopaminergic, GABAergic, and glutamatergic signaling pathways in humans and animals [61,62,63]. Studies have also indicated that chronic exposures to Mn at levels that are below the known risk threshold for manganese, could still cause behavioral, cognitive, and motor dysfunctions [14,64]. However, commercial complete dry pet food are often extruded, thus it should be also considered that extrusion treatment substantially affect nutritional quality of ingredient. The study investigating the effect of extrusion on digestibility of different blends containing *Hermetia illucens* demonstrated that extrusion can contribute to increase in organic matter digestibility [65]. Hence, it is worth investigating this relatively new source of protein in dog food more closely, as it may have consequences of a Mn excess in finished products, and thus its excess in the dog’s body and its toxic effect. On the other hand, in four other tested foods, the determined amount of Mn was lower than that declared on the label (Table 4). These four foods came from one producer and the declared amount of Mn exceeded the actual values by 42.36% to 109.07%, which indicates the lack of supplementation despite the manufacturer’s declarations. The same four foods also exceeded the values given on the label with the amount of Zn from 2.15% to 19.23% above declared values. While Mn supplementation at the declared level in these four foods does not seem necessary, the Zn content in these foods were at the lower amounts, slightly above the MRL (Table 3).

Zinc deficiency is well-known in dog nutrition and, among other things, may reduce Zn serum concentration, affect animals’ growth, cause skin lesions, behavioral problems, and compromise the immune function [66,67]. In pet food, it is common practice to supplement diets with a Zn level above the minimum requirement to prevent symptoms of deficiency [12]. Goi et al. [68] found that in the tested extruded commercial dog foods the amounts of Zn were greater than the established safe limit, and the average content of all foods was close to the recommended maximum (mean 19.02 g/100 g DM). In turn, a mean above the MLL of Zn in twenty dry complete dog foods for adult dogs and six foods for puppies (mean 32.5 and 27.6 g/100 g DM, respectively) was found in a study conducted by Pereira et al. [11]. Moreover, all tested foods for puppies exceeded MLL for Zn (range 24.8 g–31.7 g/100 g DM). In our study, the largest incompatibilities with the recommended standards concerned the amount of Zn in dog foods, where five tested foods exceeded the MLL and many of them had quantities close to the recommended maximum value (Table 3, Figure 1B). Therefore, it can be concluded that Zn in dog food is more often in excess than in a deficit. Interestingly, dog foods which exceeded the MLL of Zn, Cu, and Fe had been enriched with nutritional additives, although apparently they did not require any supplementation, since this led to exceeded MLL.

However, the level of an element in the feed is not the only factor as the bioavailability and metabolism of minerals are complex. Many factors influence the absorption and use of a mineral by the animal’s body. Intestinal absorption of trace elements such as Zn or Cu can be noticeably reduced if the diet has relatively high dietary fiber or phytate content [11,53]. As well as this, high levels of Ca in the diet can inhibit the absorption of Fe and Zn in the gastrointestinal tract [69]. Recent studies on the development of a new system for the evaluation of bioaccessible Zn in dry dog food, showed that most of the foods presented total Zn levels higher than the MLL, but this value was not surpassed if the bioaccessible Zn content was considered [70]. Therefore, it should be borne in mind that the bioavailability of trace minerals determines efficacy.

### 3.4. Relations between Elements

The quality of dog food depends not only on the amount of individual minerals consumed but also in their proportions. The dietary pattern approach should consider the role of all nutrients because ratios of nutrients in a complete diet, are more informative than each single nutrient’s contribution [71]. Since no person or pet animal consumes a single element (macro- or microelement) separately in a meal. Incorrect proportions of minerals may harm animal health and be harmful to the bioavailability of some minerals.

Dog food should maintain Ca within the recommended minimum and maximum values, as well as in the appropriate ratio to P. Nutritional guideline established the Ca:P MRL ratio at the level of 1:1 and the MNL at 2:1 [3]. Inverting this ratio may result in the inadequate absorption of Ca. An appropriate Ca:P ratio is an important factor for calcium phosphate homeostasis. One of the most common diseases in dogs associated with an improperly balanced diet is secondary hyperparathyroidism (NSHP) due to a Ca deficiency with a Ca:P ratio below 1:1. Clinical signs of this disease include micro or complete bone fractures, and the duration of the disease depends on the age of the animal [16,72]. In our study, five of the tested dry dog foods (17%) had a Ca:P ratio below the MRL (ranging from 0.42:1 to 0.98:1). Davies et al. [53] also marked mineral imbalance in tested foods for dogs and cats, where two foods had Ca:P ≤ 0.25 whereas three others had a Ca:P ratio of ≥2.5. An excess of Ca to P ratio in the dog’s diet can also lead to disease. In our study, three foods exceeded the MNL for the Ca:P ratio established by FEDIAF [3], of which two of them had a ratio >2.3. Böswald et al. [73] report that a constant Ca:P ratio of ca. 1.4:1 should be aimed at, because of the possible metabolic effects of a variation of Ca:P ratios.

Recommended ratios between other elements are not determined by any of the nutritional guidelines for dogs. Therefore, the remaining ratios have been calculated on the basis of the MRL of individual elements. It is worth noting that calculations based on the MRL of individual elements are preliminary suggestions.

The Ca:Mg ratio gained a lot of attention in recent years. Inappropriate ratio increases the risk of metabolic, inflammatory, and cardiovascular disorders. Adequate Mg levels are essential for the absorption and metabolism of calcium and vitamin D [74]. It has been proven that the Ca content of the diet affects the Mg requirement [75]. In our study, thirteen dog foods (43%) had Ca:Mg ratios below the calculated ratio on the basis of the MRL for individual elements and twenty-two dog foods (73%) had a K:Mg ratio below the MRL-based ratio.

The appropriate K level is necessary to sensitize tissues to the effects of thyroid hormones. In adult dogs, too low K level in the diet leads to decrease blood pressure and cardiac output [76]. The K excess usually results from kidney dysfunction [77]. A high calcium to potassium ratio may indicate hypothyroidism and/or a decreased cellular response to thyroxine. Long-term food intake with a high ratio of calcium to potassium may appears, among others, in chronic fatigue, depression, and tendency to being overweight. In our study, eight dog foods (27%) had K:Ca ratio above the calculated ratio on the basis of the MRL for individual elements.

Sodium deficiency can lead to increased heart rate, dehydration, increased thirst, ataxia, and lethargy [78]. The accurate concentration of Na and K in the plasma is maintained by balanced intake and excretion. In our study among the calculated minimum ratios, only the Na:K ratio was met in all tested foods. In turn, Brunetto et al. [39] examined 25 wet foods for dogs and cats, whereof 28% of tested foods exceeded 1.5 g/100 g DM of Na, which is regarded as safe by FEDIAF [3]. However, FEDIAF declares that perhaps higher levels may be safe, but no scientific data are available. Although an excess of Na is observed in dog nutrition, especially in wet foods [53], inadequate Na:K ratios are due to increased K levels rather than decreased Na levels [79].

Reduced iron bioavailability may cause anemia, manganese deficiency can cause slow or impaired growth and soft-tissue calcification [80], and zinc deficiency causes mainly skin lesions, behavioral problems, thymus atrophy, and compromised immune function [12,67]. Even if the amount of the mineral in the food is above the minimum amount recommended, deficiency symptoms may occur because of its interaction with other elements and the presence of dietary antagonists [69]. For example, Mn is effectively adsorbed in the gastrointestinal tract by the bivalent metal transporter 1 (DMT1), which is also responsible for Fe transport. DMT1 is upregulated in Fe deficiency, and Fe deficiency anemia has been associated with increased Mn levels in humans, with reports of resulting neurotoxicity [81]. This association of Mn with Fe is not fully understood in dogs, but one of the conference reports concluded that Fe deficiency, anemia, does not increase Mn concentration in blood [82]. In this work, in three of the foods we examined, the Fe:Mn ratio was below the ratio calculated on the basis of the MRL of individual elements and at the same time the ratio of Mn:Zn was significantly different from the other values—up to 4 times more than the average in all foods. Providing all these nutrients in the right amount and in the accurate proportions is the key to keeping the body healthy and in good form.

### 3.5. Heavy Metals Content

Even in trace amounts, heavy metals can cause serious problems for all organisms, and their toxicity is related to its accumulation in tissues and depends on the frequency of exposure, the amount absorbed, and the absorption channel [21]. This subject is increasingly present in the field of pet food, although the amount of available research is still limited. While the presence of heavy metals (Co, Cd, Pb, Mo, Cr, Ni) was below the detection limit in our study, several studies evaluating the content of heavy metals in pet foods have already been conducted. Duran et al. [83] detected heavy metals in commercial pet food samples available in Turkey, although their concentrations appeared to meet the regulated nutritional values by AAFCO. Fernandes et al. [18] evaluated ninety-five dry dog foods for puppies and adult dogs in Brazil, whereof the mass fractions for most of the 20 elements determined (including As, Cd, and Hg) were within the permissible limit for dog health. However, Al, Sb, and U were present in some samples above the maximum levels established for humans. Furthermore, the authors concluded that the large difference between the minimum and maximum values observed for tested elements indicates that the presence of high mass fractions can be avoided by careful ingredient selection in conjunction with good manufacturing practices. Another study [84] evaluated mercury concentrations in dry and wet pet food in the United States and observed that the type of ingredients in pet food influences the metal content, since foods with fish (tuna, shrimp, and salmon) had higher concentrations of this metal. A similar conclusion was reached by Kim et al. [19], stating that poultry-based diets for dogs had relatively lower heavy metal and As content than red meat and fish-based diets. Therefore, taking into account the research of other authors, studies that considered the presence of heavy metals in pet food and their chronic intake seem to be necessary to better understand the safe limits for dogs.

## 4. Materials and Methods

### 4.1. Sampling

The research material consisted of thirty complete dry extruded maintenance diets for adult dog in packages of 0.8, 1, and 2 kg. Dog foods were selected because of the presence or absence of cereals in the composition and also based on the availability in major pet stores and commercial suppliers in the city of Szczecin (53°25.7364′ N, 14°33.1812′ E, Poland). Key nutritional information provided on the label was recorded such as macronutrient content (percentage protein, fat, moisture, ash, and fiber, as fed) alongside the country of origin and batch number. Although not exhaustive, the selected samples provide a snapshot of European dry dog foods from different market segments.

Dog foods were assigned one of two groups depending on whether cereal was a named ingredient on the label (cereal-free foods, *n* = 17, cereal foods, *n* = 13). The composition of the main ingredients of analyzed dog foods is shown in Table 5. All samples were packaged in sealed bags. After opening, from three packages of individual dog foods samples were collected, in order to obtain one representative sample for chemical analysis. The samples were then ground into powder using a laboratory mill (KNIFETEC 1095, Foss Tecator, Höganäs, Sweden) and placed in sterile containers marked with successive symbols (no 1–30). Three measurement replication was conducted. The numbering of the thirty dog foods tested is consistent in all tables and figures.

### 4.2. Basic Composition

DM, crude protein, crude fiber, ether extract, and crude ash were determined according to AOAC [85]. Chemicals used in the analysis of crude protein, lipids, and crude fiber were purchased from Avantor Performance Materials Poland S.A., Gliwice, Poland. To determine dry matter, samples were dried at 105 °C to constant weight. Crude protein (N × 6.25) was identified by the Kjeldahl method, using a Büchi Scrubber B414 unit and a Büchi 324 distillation set (Büchi Labortechnik AG, Flawil, Switzerland). Crude fat content was assigned using traditional Soxhlet extraction method with diethyl ether. Crude fiber was determined as the residue after sequential treatment with 1.25% H_2_SO_4_ and with 1.25% NaOH using an ANKOM^220^ Fiber Analyzer (ANKOM Technology, New York, NY, USA). Crude ash was measured by burning in a muffle furnace at 580 °C for 8 h. NFE content of each diet was calculated on the basis of the assessed chemical composition. The results are expressed in g per 100 g DM.

On the basis of identified chemical composition, ME (kcal/100 g DM) of the foods was calculated, according to the equation provided by the NRC [4], using Atwater factors.

### 4.3. Macroelements Analysis

The material for macroelelement (Ca, P, K, Na, Mg) concentration analyses was subjected to digestion in concentrated sulfuric acid (H_2_SO_4_) and perchloric acid (HClO_4_). Analyses for Ca, K, Na, and Mg were performed using an atomic absorption spectrometer (Thermo Fisher Scientific iCE 3000 Series, Waltham, Massachusetts, USA). For the determination of Ca, K, and Mg the following wavelengths has been set: K: 766.5 nm; Ca: 422.6 nm; Mg: 285.2 nm. The calculation of the content of each element was started with a calibration curve, taking into account the mass of the tested portion and the dilutions used. After mineralization of the food in a solution of sulfuric (VI) acid and H_2_O_2_, phosphorus (P) analysis were performed by colorimetric method using ammonium molybdate at 470 nm [86]. The levels of P were determined using a Specol 221 apparatus spectrophotometer (Carl Zeiss Jena, Germany). The absorbance value of the sample determined spectrophotometrically, from P_2_O_5_ to the total phosphorus content was calculated according to a chemical equivalent (0.436). The reliability of the method used was confirmed by comparative studies, among others calibration curve, using the pattern series method. The results of Ca, P, K, Mg, and Na analyses were expressed in g per 100 g of DM.

### 4.4. Microelements and Heavy Metal Analysis

Material for microelements (Fe, Zn, Mn, Cu) and heavy metal (Co, Cd, Pb, Mo, Cr, Ni) concentration analyses was subjected to digestion in a nitric acid (HNO_3_) and perchloric acid (HClO_4_) mixture. Analyses were performed using an atomic absorption spectrometer (Thermo Fisher Scientific iCE 3000 Series, Waltham, Massachusetts, USA). For the determination of Fe, Mn, Zn, and Cu the following wavelengths has been set: Fe: 248.3 nm; Mn: 279.5 nm; Zn: 213.8 nm; Cu: 324.8 nm. The calculation of the content of each element was started with a calibration curve, taking into account the mass of the tested portion and the dilutions used. The results of Fe, Zn, Mn, and Cu analyses were expressed in mg per 100 g of DM.

The levels of all minerals were compared with recommendations from FEDIAF [3] considering an energy intake of 110 kcal/kg body weight (BW)^0.75^ for dogs with moderate activity (1–3 h/day).

### 4.5. Amount of Trace Elements from Feed Additives

Key nutritional information provided on the label was recorded, including, where available, the concentration of minerals added to each food (additional Cu, Fe, Mn and Zn in mg/kg). The declared levels of compounds of trace elements on the dog food labels (wherever available) were converted into the levels of actual elements (Table S1). The share of selected minerals which originated from feed additives was estimated in the total amount of determined minerals.

### 4.6. Relations between Elements

The nutritional guidelines have only established MRL and MNL for the Ca:P ratio. Recommended ratios between other elements are not outlined by nutritional guidelines. For this preliminary exploration of elements ratios the remaining ratios have been calculated on the basis of the MRL of individual elements. Ratios Ca:Mg, K:Mg, Na:K, K:Ca, Fe:Mn, Mn:Zn, and P:Zn have been calculated. However, these ratios are for illustrative purposes, supporting the concept that mineral levels are relative.

### 4.7. Statistical Analysis

One factor analysis of variance (ANOVA) for all analyzed features and a principal component analysis (PCA) were carried out using the STATISTICA 13.0 software (TIBCO Software Inc., Palo Alto, CA, USA) [87]. The significance of differences between means was assessed using the Tukey test at *p* = 0.05. According to the test results the means was divided into homogeneous groups. The means denoted by different letters differ statistically at α = 0.05 (for all columns separately). In order to compare the mineral profile of the dog foods, we determined their elemental content (Ca, P, K, Na, Mg, Fe, Zn, Mn, Cu). The percentage of a given element in the profile is expressed by an arithmetic mean converted into units on a 9-point scale. For profile comparison, Cohen’s profile similarity coefficient *r_c_* was used which was calculated from the formula [88]:

$$r_{C}=\frac{\overset{n}{\underset{i=1}{\sum }}A_{i}B_{i}+nm^{2}-m\left(\overset{n}{\underset{i=1}{\sum }}A_{i}+\overset{n}{\underset{i=1}{\sum }}B_{i}\right)}{\sqrt{\left(\overset{n}{\underset{i=1}{\sum }}A_{i}^{2}+nm^{2}-2m\overset{n}{\underset{i=1}{\sum }}A_{i}\right)\left(\overset{n}{\underset{i=1}{\sum }}\overset{2}{\underset{i}{B}}+nm^{2}-2m\overset{n}{\underset{i=1}{\sum }}B_{i}\right)}}$$

where: *A_i_*, *B_i_*—unitarized values of traits included in the compared profiles A and B; *n*—number of traits in the profile; *m*—midpoint of the ranking scale.

This coefficient value was measured in the range from −1.0 to 1.0 and its interpretation was dependent on the values: x ≥ +0.75 (high similarity); +0.75 > x > +0.30 (moderate similarity); +0.30 ≥ x ≥ −0.30 (no similarity); −0.30 > x > −0.75 (moderate dissimilarity); x ≤ −0.75 (high dissimilarity). The closer the values of *r_c_* were to boundary values (1/−1), the stronger the evaluated similarity/dissimilarity was. Inter-profile analysis was conducted using MS Office 2017.

## 5. Conclusions

The results of this study suggest that there is considerable non-compliance with current EU guidelines for pet food. Eighteen out of thirty tested dry dog foods (60%) differed from at least one recommendation established by the nutritional guidelines. The obtained results are as follows:

- All the evaluated dry dog foods met the minimum FEDIAF recommended levels for protein and fat;
- Comparative analysis of the mineral profile of foods showed differences between foods depending on the composition, which allowed to distinguish specific groups of foods—among the tested foods significant differences in the presence or absence of cereals in the composition were found, with higher P, K, Na, Fe, Zn, Mn, and Cu levels found in cereal-free foods;
- For a few individual products, mineral content often either far exceeded or did not meet the nutritional requirements, of which in two dog foods, the limit for two trace elements exceeded simultaneously and the concentration of exceeded element was supplemented with nutritional additives, which possibly led to their excess;
- Dog foods without the declared supplementation with nutritional additives met the minimum recommended levels of trace elements, and in two foods they even exceeded the legal limit;
- Diets with an insect source of animal protein had relatively high Mn content, which was supplied from raw materials, not nutritional additives; this may indicate the need for a more detailed analysis of this relatively new source of protein in dogs’ diets in order to avoid excess Mn in the finished product and thus its toxicity;
- Most of the incompatibilities concerned inappropriate mineral Ca:P ratio content (five dog foods below the minimum recommended levels and three above maximum), which may constitute a risk to the overall well-being and health of pets when they are exclusively fed with these products in the long term;
- Heavy metals (Co, Cd, Pb, Mo, Cr, Ni) levels were below detection limit in all analyzed dog foods.

Therefore, the results argue for the need to conduct more frequent, regular, more precise, and restrictive feed analyses to know the content of the elements of the raw materials before planning supplementation as well as to monitor the mineral content of finished pet food. The information provided on the dog food label should be more comprehensive and include detailed information concerning mineral content.

## Figures and Tables

**Table 1:** Nutrients (g/100 g DM) and energy value (kcal/100 g DM) content of the studied dry dog foods ^1^.

No ^2^	Dry Matter (g/100 g)	Crude Protein	Ether Extract	Crude Fiber	Crude Ash	NFE ^3^	ME ^4^
1	93.77 ^kl^	34.14 ^n^	21.39 ^p^	13.23 ^op^	7.28 ^mn^	17.74 ^a^	400.0 ^nop^
2	92.85 ^fghi^	28.14 ^ijkl^	11.61 ^cdefg^	5.59 ^cdefg^	7.78 ^o^	39.74 ^efg^	376.0 ^defghi^
3	92.86 ^ghi^	24.40 ^cd^	9.82 ^bcdef^	5.71 ^defgh^	7.74 ^o^	45.21 ^hijk^	366.8 ^cde^
4	92.45 ^cde^	29.42 ^lm^	12.26 ^efgh^	7.30 ^ghijk^	5.92 ^cd^	37.56 ^def^	378.2 ^defghi^
5	92.46 ^cde^	25.71 ^def^	8.02 ^ab^	5.83 ^defghi^	9.92 ^r^	43.00 ^gh^	347.0 ^a^
6	92.45 ^cde^	28.27 ^ijkl^	14.74 ^hijklm^	5.94 ^defghi^	6.74 ^jk^	36.77 ^de^	392.8 ^jklmnop^
7	93.60 ^k^	26.05 ^efg^	14.72 ^hijklm^	4.12 ^abcde^	6.07 ^cde^	42.65 ^gh^	407.2 ^pq^
8	96.91 ^q^	21.95 ^a^	16.98 ^lmno^	11.55 ^mno^	6.49 ^hi^	39.95 ^efg^	400.4 ^nop^
9	94.51 ^n^	22.73 ^ab^	9.82 ^bcdef^	8.07 ^hijkl^	4.93 ^a^	48.96 ^jklm^	375.1 ^cdefghi^
10	96.82 ^q^	29.27 ^klm^	12.62 ^ghi^	7.30 ^ghijk^	5.87 ^c^	41.78 ^fgh^	397.7 ^lmnop^
11	95.87 ^o^	38.12 ^o^	17.98 ^o^	11.76 ^no^	7.71 ^o^	20.32 ^a^	395.5 ^klmnop^
12	97.39 ^r^	38.07 ^o^	16.49 ^klmno^	4.32 ^bcdef^	9.88 ^r^	28.64 ^b^	415.2 ^q^
13	96.34 ^p^	25.14 ^cde^	11.81 ^efg^	6.59 ^fghij^	7.19 ^lmn^	45.61 ^hijk^	389.3 ^ijklmno^
14	95.95 ^o^	24.29 ^cd^	11.84 ^efg^	6.52 ^efghij^	8.56 ^p^	44.75 ^hij^	382.7 ^fghijk^
15	93.48 ^jk^	28.91 ^jklm^	8.89 ^abc^	6.39 ^efghij^	7.08 ^lm^	42.23 ^gh^	364.5 ^bcd^
16	92.56 ^defg^	23.90 ^bc^	6.31 ^a^	6.35 ^efghij^	6.54 ^hij^	49.46 ^klm^	350.2 ^ab^
17	94.14 ^m^	27.92 ^ijk^	6.76 ^a^	3.22 ^abc^	7.17 ^lmn^	49.07 ^klm^	368.8 ^cdef^
18	92.82 ^fghi^	21.40 ^a^	12.33 ^fgh^	8.20 ^ijkl^	7.31 ^n^	43.59 ^ghi^	370.9 ^cdefg^
19	92.15 ^c^	26.26 ^efg^	15.07 ^ijklmn^	7.84 ^ghijkl^	7.30 ^n^	35.68 ^de^	383.4 ^ghijkl^
20	92.37 ^cd^	30.25 ^m^	11.65 ^defg^	8.52 ^jkl^	8.05 ^p^	33.91 ^cd^	361.4 ^abc^
21	91.11 ^a^	38.97 ^o^	16.47 ^klmno^	7.59 ^ghijkl^	7,82 ^o^	20.27 ^a^	385.2 ^ghijklm^
22	92.85 ^fghi^	34.23 ^n^	18.21 ^o^	15.14 ^p^	6.37 ^fgh^	18.92 ^a^	376.4 ^defghi^
23	93.15 ^ij^	34.74 ^n^	17.25 ^mno^	4.24 ^bcdef^	9.10 ^q^	27.83 ^b^	405.5 ^pq^
24	91.07 ^a^	30.55 ^m^	7.08 ^a^	4.86 ^bcdef^	7.89 ^o^	53.17 ^n^	398.58 ^lmnop^
25	92.53 ^def^	29.20 ^jklm^	16.81 ^lmno^	11.90 ^no^	4.80 ^a^	29.83 ^bc^	387.3 ^hijklmn^
26	93.57 ^k^	27.75 ^hij^	13.51 ^ghij^	9.13 ^klm^	6.68 ^ijk^	36.50 ^de^	378.6 ^defghij^
27	92.68 ^defgh^	26.39 ^efgh^	13.90 ^ghijk^	2.90 ^ab^	6.43 ^gh^	43.07 ^gh^	402.9 ^opq^
28	91.54 ^b^	22.58 ^ab^	9.55 ^bcde^	3.59 ^abcd^	5.42 ^b^	50.41 ^lmn^	377.9 ^defghi^
29	91.64 ^b^	35.41 ^n^	15.77 ^jklmno^	7.75 ^ghijkl^	7.10 ^mno^	25.62 ^b^	386.0 ^hijklmn^
30	97.55 ^r^	22.41 ^a^	17.62 ^no^	13.38 ^op^	6.66 ^ijk^	37.49 ^def^	398.2 ^mnop^
MRL ^5^		18.00	5.50	-	-	-	-

^1^ Means with at least one same letter in the superscripts (a, b, c, …) not differ statistically at *P* = 0.05 (for all columns separately); ^2^ The main ingredients of analyzed dog foods and division into cereal and cereal-free foods are shown in Table 5; ^3^ NFE, nitrogen free extract; ^4^ ME, metabolizable energy; ^5^ MRL, minimum recommended level [3].

**Table 2:** Macroelements (g/100 g DM) contents of the studied dry dog foods ^1^.

No ^2^	Ca	P	K	Na	Mg
1	0.451 ^a^	1.075 ^o^	1.349 ^r^	0.660 ^s^	0.094 ^a^
2	0.759 ^cde^	0.725 ^efg^	0.953 ^kl^	0.363 ^d^	0.132 ^h^
3	1.296 ^m^	0.793 ^ij^	0.882 ^j^	0.407 ^f^	0.120 ^cd^
4	0.751 ^cde^	0.535 ^b^	0.831 ^i^	0.313 ^b^	0.097 ^a^
5	1.640 ^pq^	0.856 ^kl^	1.082 ^o^	0.431 ^hi^	0.152 ^k^
6	1.103 ^jkl^	0.535 ^b^	0.706 ^f^	0.450 ^l^	0.167 ^m^
7	0.995 ^ghijk^	0.717 ^ef^	0.794 ^h^	0.396 ^e^	0.094 ^a^
8	0.857 ^defg^	0.475 ^a^	1.182 ^q^	0.482 ^m^	0.184 ^o^
9	0.636 ^bc^	0.599 ^c^	0.939 ^k^	0.251 ^a^	0.172 ^n^
10	0.882 ^efgh^	0.897 ^lm^	0.588 ^ab^	0.395 ^e^	0.182 ^o^
11	0.964 ^fghij^	0.818 ^jk^	0.883 ^j^	0.687 ^t^	0.152 ^k^
12	1.522 ^op^	0.660 ^d^	1.004 ^m^	0.834 ^u^	0.181 ^o^
13	1.109 ^kl^	0.765 ^ghi^	0.638 ^c^	0.535 ^o^	0.138 ^i^
14	1.476 ^no^	0.777 ^i^	0.610 ^b^	0.504 ^n^	0.126 ^fg^
15	0.561 ^ab^	0.674 ^d^	0.710 ^f^	0.445 ^kl^	0.218 ^r^
16	0.855 ^def^	0.548 ^b^	0.849 ^i^	0.533 ^o^	0.112 ^b^
17	1.145 ^l^	0.928 ^mn^	0.966 ^l^	0.425 ^h^	0.128 ^gh^
18	1.554 ^op^	0.900 ^mn^	0.652 ^cd^	0.438 ^jk^	0.120 ^d^
19	1.284 ^m^	0.754 ^fghi^	0.710 ^f^	0.417 ^g^	0.139 ^i^
20	1.605 ^op^	0.896 ^lm^	0.827 ^i^	0.437 ^ij^	0.125 ^efg^
21	1.030 ^ijkl^	0.938 ^n^	1.001 ^m^	0.828 ^u^	0.189 ^p^
22	0.741 ^cd^	0.537 ^b^	0.997 ^m^	0.631 ^r^	0.159 ^m^
23	1.746 ^q^	0.754 ^fghi^	0.832 ^i^	0.503 ^n^	0.149 ^jk^
24	1.543 ^op^	0.843 ^k^	0.744 ^g^	0.575 ^q^	0.148 ^j^
25	0.539 ^ab^	0.735 ^fgh^	0.833 ^i^	0.417 ^g^	0.121 ^de^
26	1.315 ^m^	0.690 ^de^	0.676 ^e^	0.436 ^ij^	0.116 ^bc^
27	1.345 ^mn^	0.914 ^mn^	0.663 ^de^	0.406 ^f^	0.124 ^def^
28	0.915 ^fghi^	0.767 ^hi^	0.568 ^a^	0.328 ^c^	0.124 ^def^
29	0.952 ^fghi^	1.204 ^p^	1.050 ^n^	0.564 ^p^	0.163 ^m^
30	1.018 ^hijkl^	0.549 ^b^	1.149 ^p^	0.430 ^hi^	0.200 ^q^
Contrast					
Cereal-free foods	1.083 ^a^	0.771 ^b^	0.964 ^b^	0.527 ^b^	0.145 ^a^
Cereal foods	1.091 ^a^	0.750 ^a^	0.713 ^a^	0.428 ^a^	0.144 ^a^
MRL ^3^	0.500	0.400	0.500	0.100	0.07
MNL ^4^	2.500	1.600	-	-	-

^1^ Means with at least one same letter in the superscripts (a, b, c, …) did not differ statistically at *P* = 0.05 (for all columns separately); ^2^ the main ingredients of analyzed dog foods and division into cereal and cereal-free foods are shown in Table 5; ^3^ MRL, minimum recommended level [3]; ^4^ MNL, maximum nutritional limit [3].

**Table 3:** Trace elements (mg/100 g DM) contents of the studied dry dog foods ^1^.

No ^2^	Microelements				Heavy Metals ^5^					
	Cu	Fe	Mn	Zn	Co	Cd	Pb	Mo	Cr	Ni
1	2.374 ^kl^	55.99 ^s^	4.035 ^l^	21.22 ^hi^	BDL	BDL	BDL	BDL	BDL	BDL
2	2.501 ^mn^	99.56 ^x^	6.505 ^p^	11.11 ^abcd^	BDL	BDL	BDL	BDL	BDL	BDL
3	2.157 ^j^	49.47 ^p^	4.429 ^m^	9.52 ^a^	BDL	BDL	BDL	BDL	BDL	BDL
4	2.604 ^o^	56.25 ^s^	4.589 ^n^	10.45 ^ab^	BDL	BDL	BDL	BDL	BDL	BDL
5	1.277 ^a^	72.61 ^u^	5.936 ^o^	10.58 ^abc^	BDL	BDL	BDL	BDL	BDL	BDL
6	1.218 ^a^	29.25 ^f^	3.142 ^i^	13.02 ^abcde^	BDL	BDL	BDL	BDL	BDL	BDL
7	2.578 ^no^	33.60 ^i^	4.526 ^mn^	15.72 ^def^	BDL	BDL	BDL	BDL	BDL	BDL
8	2.142 ^j^	44.05 ^m^	21.210 ^v^	15.90 ^defg^	BDL	BDL	BDL	BDL	BDL	BDL
9	1.904 ^gh^	29.79 ^f^	9.522 ^t^	10.98 ^abcd^	BDL	BDL	BDL	BDL	BDL	BDL
10	1.629 ^ef^	31.93 ^gh^	3.330 ^j^	12.92 ^abcde^	BDL	BDL	BDL	BDL	BDL	BDL
11	1.693 ^f^	84.60 ^w^	7.904 ^s^	18.77 ^fghi^	BDL	BDL	BDL	BDL	BDL	BDL
12	2.825 ^p^	53.86 ^r^	7.042 ^q^	40.43 ^k^	BDL	BDL	BDL	BDL	BDL	BDL
13	2.290 ^k^	51.47 ^q^	7.127 ^q^	30.81 ^j^	BDL	BDL	BDL	BDL	BDL	BDL
14	1.583 ^de^	77.89 ^v^	7.137 ^q^	17.22 ^efgh^	BDL	BDL	BDL	BDL	BDL	BDL
15	1.497 ^cd^	63.65 ^t^	7.788 ^r^	17.97 ^fghi^	BDL	BDL	BDL	BDL	BDL	BDL
16	1.677 ^f^	45.90 ^n^	3.125 ^i^	17.02 ^efgh^	BDL	BDL	BDL	BDL	BDL	BDL
17	1.855 ^g^	29.33 ^f^	2.594 ^g^	15.48 ^cdef^	BDL	BDL	BDL	BDL	BDL	BDL
18	1.406 ^bc^	31.47 ^g^	3.600 ^k^	18.17 ^fghi^	BDL	BDL	BDL	BDL	BDL	BDL
19	2.002 ^i^	48.09 ^o^	2.350 ^f^	20.70 ^ghi^	BDL	BDL	BDL	BDL	BDL	BDL
20	2.927 ^q^	42.81 ^l^	2.071 ^d^	29.08 ^j^	BDL	BDL	BDL	BDL	BDL	BDL
21	2.288 ^k^	14.37 ^b^	0.998 ^a^	21.51 ^hi^	BDL	BDL	BDL	BDL	BDL	BDL
22	2.416 ^lm^	28.31 ^e^	2.550 ^g^	31.54 ^j^	BDL	BDL	BDL	BDL	BDL	BDL
23	1.683 ^f^	45.89 ^n^	2.916 ^h^	17.73 ^efghi^	BDL	BDL	BDL	BDL	BDL	BDL
24	2.162 ^j^	40.70 ^k^	2.508 ^g^	27.56 ^j^	BDL	BDL	BDL	BDL	BDL	BDL
25	1.634 ^ef^	26.91 ^d^	1.685 ^c^	20.98 ^hi^	BDL	BDL	BDL	BDL	BDL	BDL
26	1.716 ^f^	32.11 ^h^	2.247 ^e^	18.46 ^fghi^	BDL	BDL	BDL	BDL	BDL	BDL
27	1.685 ^f^	29.46 ^f^	2.199 ^e^	18.42 ^fghi^	BDL	BDL	BDL	BDL	BDL	BDL
28	1.395 ^b^	18.23 ^c^	1.374 ^b^	15.12 ^bcdef^	BDL	BDL	BDL	BDL	BDL	BDL
29	1.922 ^ghi^	12.75 ^a^	1.092 ^a^	22.53 ^i^	BDL	BDL	BDL	BDL	BDL	BDL
30	1.960 ^hi^	35.72 ^j^	18.300 ^u^	13.87 ^abcdef^	BDL	BDL	BDL	BDL	BDL	BDL
Contrast										
Cereal-free foods	2.185 ^b^	46.91 ^b^	5.782 ^b^	19.91 ^b^	-	-	-	-	-	-
Cereal foods	1.681 ^a^	39.89 ^a^	4.272 ^a^	17.41 ^a^	-	-	-	-	-	-
MRL ^3^	0.720	3.60	0.580	7.20	-	-	-	-	-	-
MLL ^4^	2.800	68.18	17.000	22.70	-	-	-	-	-	-

^1^ Means with at least one same letter in the superscripts (a, b, c, …) did not differ statistically at *P* = 0.05 (for all columns separately); ^2^ the main ingredients of analyzed dog foods and division into cereal and cereal-free foods are shown in Table 5; ^3^ MRL, minimum recommended level [3]; ^4^ MLL, maximum legal limit [3]; ^5^ BDL—below detection limit.

**Table 4:** Percentage of total determined trace element content sourced by the labeled additives ^1^.

No	Cu	Fe	Mn	Zn
1	17.48	3.11	34.30	9.33
2	66.36	5.74	142.36	102.15
3	76.95	11.55	209.07	119.23
4	63.74	10.16	201.78	108.64
5	129.94	7.87	155.98	107.35
6	NDL	NDL	NDL	NDL
7	16.46	7.61	66.54	45.43
8	50.74	NDL	NDL	68.37
9	20.80	5.88	14.01	17.69
10	NDL	NDL	NDL	NDL
11	12.53	8.68	68.59	57.06
12	8.01	13.63	75.76	26.49
13	9.88	14.26	76.07	15.77
14	14.29	9.42	75.96	28.20
15	14.17	11.53	69.05	59.60
16	16.87	8.75	68.84	70.84
17	15.24	13.70	82.95	72.09
18	20.12	12.77	59.75	66.32
19	15.89	NDL	NDL	44.10
20	10.87	NDL	NDL	50.24
21	54.64	NDL	NDL	42.45
22	45.00	38.39	42.63	48.25
23	64.58	23.69	37.27	85.82
24	12.80	8.78	33.56	20.12
25	16.93	13.27	49.95	26.42
26	16.30	11.25	8.89	27.10
27	16.59	12.26	9.09	27.16
28	19.83	NDL	NDL	66.44
29	15.05	NDL	NDL	40.53
30	14.11	NDL	NDL	62.98

^1^ NDL—not declared in the label.

**Table 5:** Comparative analysis of the mineral profile of the studied dry dog foods.

No	1	2	3	4	5	6	7	8	9	10	11	12	13	14	15	The Main Ingredients
1	-	-	-	-	-	-	-	-	-	-	-	-	-	-	-	beef, sweet potatoes, beans, beef fat
2	0.31	-	-	-	-	-	-	-	-	-	-	-	-	-	-	chicken, sweet potatoes, peas, potatoes, chicken fat
3	0.18	0.57	-	-	-	-	-	-	-	-	-	-	-	-	-	pork, sweet potatoes, peas, potatoes, pork fat
4	0.28	0.77	0.77	-	-	-	-	-	-	-	-	-	-	-	-	beef, sweet potatoes, potatoes, peas, beef fat
5	−0.09	0.25	0.59	0.11	-	-	-	-	-	-	-	-	-	-	-	lamb, sweet potatoes, peas, potatoes, lamb fat
6	−0.28	0.09	0.54	0.39	0.49	-	-	-	-	-	-	-	-	-	-	chicken, brown rice, chicken fat
7	0.32	0.49	0.81	0.90	0.04	0.37	-	-	-	-	-	-	-	-	-	dried salmon, potatoes, salmon protein, chicken fat
8	−0.21	0.09	−0.11	0.15	−0.09	0.11	−0.10	-	-	-	-	-	-	-	-	potatoes, *Hermetia illucens,* poultry fat
9	−0.04	0.40	0.48	0.62	0.19	0.66	0.46	0.55	-	-	-	-	-	-	-	*Hermetia illucens*, oats, potatoes, corn, peas, insects oil
10	−0.16	0.16	0.52	0.34	0.31	0.79	0.38	−0.15	0.63	-	-	-	-	-	-	salmon, brown rice, chicken fat
11	0.17	0.40	0.16	0.09	0.40	0.28	−0.11	−0.21	−0.11	0.20	-	-	-	-	-	chicken, potatoes, dried peas, animal fat
12	−0.13	−0.37	−0.38	−0.32	−0.41	−0.32	−0.20	−0.18	−0.58	−0.47	−0.17	-	-	-	-	salmon, potatoes, peas, animal fat
13	−0.19	0.03	0.18	0.35	−0.35	0.23	0.51	−0.50	−0.08	0.29	−0.03	0.30	-	-	-	salmon, rice, animal fat
14	−0.31	0.32	0.59	0.33	0.61	0.54	0.32	−0.38	0.03	0.43	0.55	−0.28	0.41	-	-	lamb, rice, animal fat
15	−0.31	0.25	0.03	0.15	0.14	0.64	−0.09	0.24	0.62	0.70	0.40	−0.36	0.05	0.20	-	chicken, corn, rice, wheat, vegetable fiber, animal fat
16	0.24	0.39	0.64	0.72	0.30	0.76	0.67	0.00	0.52	0.48	0.41	−0.26	0.36	0.50	0.32	barley, salmon, rabbit, whole grain oat flour, potato flakes, poultry fat
17	0.36	0.20	0.81	0.50	0.51	0.59	0.68	−0.29	0.47	0.64	0.01	−0.41	0.12	0.34	0.05	duck, corn flour, rice flour, salmon, liver, sugar beet molasses, poultry fat
18	−0.16	−0.06	0.67	0.26	0.57	0.69	0.48	−0.45	0.20	0.65	0.12	−0.31	0.40	0.74	0.08	lamb, rice, corn, poultry fat
19	−0.13	0.31	0.77	0.60	0.40	0.71	0.70	−0.41	0.37	0.69	0.13	−0.12	0.64	0.70	0.25	lamb, oats, beef, pork, lamb fat, lentils, peas
20	0.07	0.10	0.47	0.35	−0.04	−0.06	0.60	−0.54	−0.16	0.06	−0.37	0.40	0.60	0.19	−0.46	lamb, peas, lentils, lamb liver, lamb fat
21	0.29	−0.31	0.08	−0.12	−0.10	0.22	0.09	−0.22	−0.02	0.32	0.05	0.41	0.08	−0.25	0.06	sardines, mackerel, hake, flounder, redfish, sole, herring, cod, blue whiting, herring oil, red lentils, green lentils, green peas, chickpeas, peas
22	0.25	−0.03	0.01	0.32	−0.41	0.28	0.36	0.06	0.24	0.10	−0.12	0.49	0.42	−0.31	0.15	white fish, herring, salmon, salmon oil, peas, potato flakes
23	−0.29	0.00	0.68	0.21	0.73	0.68	0.34	−0.32	0.13	0.48	0.18	0.00	0.25	0.71	0.06	turkey, rabbit, peas, pork fat, potatoes
24	−0.17	−0.19	0.42	0.11	0.18	0.37	0.40	−0.65	−0.19	0.36	−0.04	0.42	0.68	0.47	−0.14	lamb, potato flakes, peas, chicken protein, *Saccharomyces cerevisiae*, salmon oil
25	0.35	0.24	0.50	0.61	0.10	0.68	0.65	−0.12	0.63	0.66	0.14	−0.42	0.36	0.22	0.41	white fish, potato flakes, peas, animal fat, salmon, chicken
26	−0.08	0.15	0.77	0.58	0.42	0.79	0.72	−0.29	0.40	0.65	0.12	−0.22	0.56	0.69	0.17	chicken, rice, peas, animal fat, beef
27	−0.04	0.06	0.74	0.41	0.46	0.68	0.63	−0.48	0.32	0.74	0.06	−0.32	0.49	0.65	0.13	chicken, rice, peas, animal fat, beef
28	−0.02	0.16	0.66	0.55	0.33	0.84	0.65	−0.20	0.62	0.85	0.11	−0.49	0.45	0.55	0.44	poultry, salmon, millet, barley, corn, rice, potatoes, dried beet pulp, animal fat
29	0.46	−0.25	0.23	−0.08	0.11	0.20	0.20	−0.44	0.12	0.49	−0.12	−0.10	−0.02	−0.19	0.01	herring, sardines, flounder, cod, hake, green peas, red lentils, chickpeas, green lentils, red banded redfish, pinto beans, peas, pollock oil
30	−0.33	0.02	0.02	0.10	0.13	0.31	−0.10	0.94	0.67	0.10	−0.24	−0.25	−0.53	−0.29	0.37	potatoes, *Hermetia illucens*, poultry fat
No	16	17	18	19	20	21	22	23	24	25	26	27	28	29	**30**	**The Main Ingredients**
16	-	-	-	-	-	-	-	-	-	-	-	-	-	-	-	barley, salmon, rabbit, whole grain oat flour, potato flakes, poultry fat
17	0.61	-	-	-	-	-	-	-	-	-	-	-	-	-	-	duck, corn flour, rice flour, salmon, liver, sugar beet molasses, poultry fat
18	0.54	0.76	-	-	-	-	-	-	-	-	-	-	-	-	-	lamb, rice, corn, poultry fat
19	0.67	0.71	0.82	-	-	-	-	-	-	-	-	-	-	-	-	lamb, oats, beef, pork, lamb fat, lentils, peas
20	0.08	0.40	0.38	0.60	-	-	-	-	-	-	-	-	-	-	-	lamb, peas, lentils, lamb liver, lamb fat
21	0.14	0.36	0.13	0.15	0.21	-	-	-	-	-	-	-	-	-	-	sardines, mackerel, hake, flounder, redfish, sole, herring, cod, blue whiting, herring oil, red lentils, green lentils, green peas, chickpeas, peas
22	0.48	0.16	−0.09	0.26	0.26	0.56	-	-	-	-	-	-	-	-	-	white fish, herring, salmon, salmon oil, peas, potato flakes
23	0.47	0.63	0.84	0.79	0.44	0.23	0.01	-	-	-	-	-	-	-	-	turkey, rabbit, peas, pork fat, potatoes
24	0.29	0.47	0.69	0.76	0.77	0.46	0.31	0.76	-	-	-	-	-	-	-	lamb, potato flakes, peas, chicken protein, *Saccharomyces cerevisiae*, salmon oil
25	0.83	0.72	0.52	0.62	0.09	0.24	0.51	0.26	0.24	-	-	-	-	-	-	white fish, potato flakes, peas, animal fat, salmon, chicken
26	0.80	0.77	0.90	0.93	0.45	0.16	0.26	0.79	0.69	0.72	-	-	-	-	-	chicken, rice, peas, animal fat, beef
27	0.59	0.85	0.96	0.89	0.48	0.21	0.06	0.77	0.71	0.66	0.93	-	-	-	-	chicken, rice, peas, animal fat, beef
28	0.76	0.79	0.83	0.82	0.20	0.16	0.22	0.57	0.46	0.87	0.90	0.90	-	-	-	poultry, salmon, millet, barley, corn, rice, potatoes, dried beet pulp, animal fat
29	0.11	0.70	0.39	0.26	0.25	0.73	0.24	0.23	0.38	0.47	0.27	0.50	0.41	-	-	herring, sardines, flounder, cod, hake, green peas, red lentils, chickpeas, green lentils, red banded redfish, pinto beans, peas, pollock oil
30	0.02	−0.08	−0.24	−0.23	−0.48	−0.09	0.01	−0.07	−0.49	−0.05	−0.14	−0.26	−0.02	−0.24	-	potatoes, *Hermetia illucens*, poultry fat

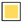
—cereal foods; 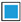
—cereal-free foods; 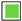
—x ≥ +0.75 (high similarity); 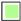
—+0.75 > x > +0.30 (moderate similarity); 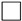
—+0.30 ≥ x ≥ -0.30 (no similarity); 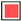
—−0.30 > x > −0.75 (moderate dissimilarity); 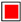
—x ≤ −0.75 (high dissimilarity).

**Table 6:** Relations between elements in the analyzed dry dog foods and comparison with ratios calculated on the basis of the FEDIAF minimum recommended levels of individual elements.

No	Ca:Mg	K:Mg	Na:K	K:Ca	Fe:Mn	Mn:Zn	P:Zn
1	4.80:1	14.35:1	0.49:1	2.99:1	13.88:1	0.19:1	50.66:1
2	5.75:1	7.22:1	0.38:1	1.26:1	15.31:1	0.59:1	65.26:1
3	10.80:1	7.35:1	0.46:1	0.68:1	11.17:1	0.47:1	83.30:1
4	7.74:1	8.57:1	0.38:1	1.11:1	12.26:1	0.44:1	51.20:1
5	10.79:1	7.12:1	0.40:1	0.66:1	12.23:1	0.56:1	80.91:1
6	6.60:1	4.23:1	0.64:1	0.64:1	9.31:1	0.24:1	41.09:1
7	10.59:1	8.45:1	0.50:1	0.80:1	7.42:1	0.29:1	45.61:1
8	4.66:1	6.42:1	0.41:1	1.38:1	2.08:1	1.33:1	29.87:1
9	3.70:1	5.46:1	0.27:1	1.48:1	3.13:1	0.87:1	54.55:1
10	4.85:1	3.23:1	0.67:1	0.67:1	9.59:1	0.26:1	69.43:1
11	6.34:1	5.81:1	0.78:1	0.92:1	10.70:1	0.42:1	43.58:1
12	8.41:1	5.55:1	0.83:1	0.66:1	7.65:1	0.17:1	16.32:1
13	8.04:1	4.62:1	0.84:1	0.58:1	7.22:1	0.23:1	24.83:1
14	11.71:1	4.84:1	0.83:1	0.41:1	10.91:1	0.41:1	45.12:1
15	2.57:1	3.26:1	0.63:1	1.27:1	8.17:1	0.43:1	37.51:1
16	7.63:1	7.58:1	0.63:1	0.99:1	14.69:1	0.18:1	32.20:1
17	8.95:1	7.55:1	0.44:1	0.84:1	11.31:1	0.17:1	59.95:1
18	12.95:1	5.43:1	0.67:1	0.42:1	8.74:1	0.20:1	49.53:1
19	9.24:1	5.11:1	0.59:1	0.55:1	20.46:1	0.11:1	36.43:1
20	12.84:1	6.62:1	0.53:1	0.52:1	20.67:1	0.07:1	30.81:1
21	5.45:1	5.30:1	0.83:1	0.97:1	14.40:1	0.05:1	43.61:1
22	4.66:1	6.27:1	0.63:1	1.35:1	11.10:1	0.08:1	17.03:1
23	11.72:1	5.58:1	0.60:1	0.48:1	15.74:1	0.16:1	42.53:1
24	10.43:1	5.03:1	0.77:1	0.48:1	16.23:1	0.09:1	30.59:1
25	4.45:1	6.88:1	0.50:1	1.55:1	15.97:1	0.08:1	35.03:1
26	11.34:1	5.83:1	0.64:1	0.51:1	14.29:1	0.12:1	37.38:1
27	10.85:1	5.35:1	0.61:1	0.49:1	13.40:1	0.12:1	49.62:1
28	7.38:1	4.58:1	0.58:1	0.62:1	13.27:1	0.09:1	50.73:1
29	5.84:1	6.44:1	0.54:1	1.10:1	11.68:1	0.05:1	53.44:1
30	5.09:1	5.75:1	0.37:1	1.13:1	1.95:1	1.32:1	39.58:1
Mean	7.87:1	6.19:1	0.58:1	0.92:1	11.50:1	0.33:1	44.92:1
According to MRL ^1^	7.14:1	7.14:1	0.20:1	1:1	6.21:1	0.08:1	55.56:1

^1^ MRL, minimum recommended level [3].

## Supplementary Materials

Table S1: Declarations of mineral additives as presented on the label with results of conversion into actual amounts (mg/kg feed); Table S2: Declarations of macroelement content under “Analytical constituents” presented on the label (g/kg feed).

## Glossary

MRL, minimum recommended level, the quantity of a nutrient that must be supplied to an animal in order to satisfy its metabolic needs; MNL, maximum nutritional limit, the highest level that should not cause any harmful effects; MLL, maximum legal limit, mandatory and the highest legally permitted amount of the nutrient.
