# Supplementary material for: Mineral Composition of Cereal and Cereal-Free Dry Dog Foods versus Nutritional Guidelines
Source: Molecules. 2020 Nov 6;25(21):5173. doi: 10.3390/molecules25215173 (PMC7664208; doi:10.3390/molecules25215173)
Supplement: Supplementary file 1 [file molecules-25-05173-s001.pdf]

molecules

Supplementary Materials

Mineral Composition of Cereal and Cereal-Free Dry Dog Foods versus Nutritional Guidelines

Katarzyna Kazimierska [1,*], Wioletta Biel [1] and Robert Witkowicz [2]

[1] Department of Monogastric Animal Sciences, Division of Animal Nutrition and Food, West Pomeranian University of Technology in Szczecin, 29 Klemensa Janickiego, 71270 Szczecin, Poland; katarzyna.kazimierska@zut.edu.pl; wioletta.biel@zut.edu.pl
[2] Department of Agroecology and Crop Production, University of Agriculture in Krakow, 21 Mickiewicza, 31120 Krakow, Poland; robert.witkowicz@urk.edu.pl
* Correspondence: katarzyna.kazimierska@zut.edu.pl; Tel.: +48-91-449-6841

Table 1. Declarations of mineral additives as presented on the label with results of conversion into actual amounts (mg/kg feed) [1].

Product	Microelement	Nutritional additives		Amount declared under heading "Analytical constituents"
		-Label declaration under heading "Additives"	Corresponding to actual amount of the element	
1	Cu	15.00 $CuSO_4 \cdot 5H_2O$	3.82	NDL
	Fe	50.00 $FeSO_4 \cdot H_2O$	16.00	NDL
	Mn	35.00 $MnSO_4$	12.73	NDL
	Zn	50.00 $ZnSO_4 \cdot H_2O$	18.21	NDL
2	Cu	60.00 $CuSO_4 \cdot 5H_2O$	15.27	NDL
	Fe	160.00 $FeSO_4 \cdot H_2O$	52.58	NDL
	Mn	110.00 MnO	85.19	NDL
	Zn	130.00 ZnO	104.44	NDL
3	Cu	60.00 $CuSO_4 \cdot 5H_2O$	15.27	NDL
	Fe	160.00 $FeSO_4 \cdot H_2O$	52.58	NDL
	Mn	110.00 MnO	85.19	NDL
	Zn	130.00 ZnO	104.44	NDL
4	Cu	60.00 $CuSO_4 \cdot 5H_2O$	15.27	NDL
	Fe	160.00 $FeSO_4 \cdot H_2O$	52.58	NDL
	Mn	110.00 MnO	85.19	NDL
	Zn	130.00 ZnO	104.44	NDL
5	Cu	60.00 $CuSO_4 \cdot 5H_2O$	15.27	NDL
	Fe	160.00 $FeSO_4 \cdot H_2O$	52.58	NDL
	Mn	110.00 MnO	85.19	NDL
	Zn	130.00 ZnO	104.44	NDL
6	Cu	NDL	–	22.00
	Fe	NDL	–	237.00
	Mn	NDL	–	29.30
	Zn	NDL	–	162.00
7	Cu	15.00 $CuSO_4 \cdot 5H_2O$	3.82	NDL
	Fe	70.00 $FeSO_4 \cdot H_2O$	23.01	NDL
	Mn	35.00 MnO	27.11	NDL
	Zn	80.00 ZnO	64.27	NDL
8	Cu	10.00 Cu	10.00	NDL
	Fe	NDL	–	NDL

	Mn	NDL	–	NDL
	Zn	100.00 Zn	100.00	NDL
9	Cu	14.00 $CuSO_4 \cdot 5H_2O$	3.56	NDL
	Fe	48.00 $FeSO_4 \cdot H_2O$	15.78	NDL
	Mn	33.00 $MnSO_4$	12.01	NDL
	Zn	48.00 $ZnSO_4 \cdot H_2O$	17.49	NDL
10	Cu	NDL	–	16.00
	Fe	NDL	–	144.00
	Mn	NDL	–	32.00
	Zn	NDL	–	144.00
11	Cu	7.50 $CuSO_4 \cdot 5H_2O$	1.91	NDL
	Fe	201.00 $FeSO_4 \cdot H_2O$	66.06	NDL
	Mn	63.00 MnO	48.79	NDL
	Zn	120.00 ZnO	96.41	NDL
12	Cu	8.00 $CuSO_4 \cdot 5H_2O$	2.04	NDL
	Fe	201.00 $FeSO_4 \cdot H_2O$	66.06	NDL
	Mn	62.00 MnO	48.02	NDL
	Zn	120.00 ZnO	96.41	NDL
13	Cu	8.00 $CuSO_4 \cdot 5H_2O$	2.04	NDL
	Fe	201.00 $FeSO_4 \cdot H_2O$	66.06	NDL
	Mn	63.00 MnO	48.79	NDL
	Zn	120.00 $ZnSO_4 \cdot H_2O$	43.71	NDL
14	Cu	8.00 $CuSO_4 \cdot 5H_2O$	2.04	NDL
	Fe	201.00 $FeSO_4 \cdot H_2O$	66.06	NDL
	Mn	63.00 MnO	48.79	NDL
	Zn	120.00 $ZnSO_4 \cdot H_2O$	43.71	NDL
15	Cu	7.50 $CuSO_4 \cdot 5H_2O$	1.91	NDL
	Fe	201.00 $FeSO_4 \cdot H_2O$	66.06	NDL
	Mn	62.50 MnO	48.40	NDL
	Zn	120.00 ZnO	96.41	NDL
16	Cu	10.00 $CuSO_4 \cdot 5H_2O$	2.55	NDL
	Fe	110.00 $FeSO_4 \cdot H_2O$	36.15	NDL
	Mn	25.00 MnO	19.36	NDL
	Zn	135.00 ZnO	108.46	NDL
17	Cu	10.00 $CuSO_4 \cdot 5H_2O$	2.55	NDL
	Fe	110.00 $FeSO_4 \cdot H_2O$	36.15	NDL
	Mn	25.00 MnO	19.36	NDL
	Zn	125.00 ZnO	100.43	NDL
18	Cu	10.00 $CuSO_4 \cdot 5H_2O$	2.55	NDL
	Fe	110.00 $FeSO_4 \cdot H_2O$	36.15	NDL
	Mn	25.00 MnO	19.36	NDL
	Zn	135.00 ZnO	108.46	NDL
19	Cu	11.00 $CuSO_4 \cdot 5H_2O$	2.80	15.00
	Fe	NDL	–	290.00
	Mn	NDL	–	25.00
	Zn	100.00 ZnO	80.34	210.00
20	Cu	11.00 $CuSO_4 \cdot 5H_2O$	2.80	20.00
	Fe	NDL	–	250.00
	Mn	NDL	–	21.00
	Zn	160.00 ZnO	128.55	230.00
21	Cu	11.00 Cu	11.00	NDL

	Fe	NDL	–	NDL
	Mn	NDL	–	NDL
	Zn	100.00 ZnO	80.34	NDL
22	Cu	10.00 Cu	10.00	NDL
	Fe	100.00 Fe	100.00	NDL
	Mn	10.00 Mn	10.00	NDL
	Zn	140.00 Zn	140.00	NDL
23	Cu	10.00 Cu	10.00	NDL
	Fe	100.00 Fe	100.00	NDL
	Mn	10.00 Mn	10.00	NDL
	Zn	140.00 Zn	140.00	NDL
24	Cu	10.00 $CuSO_4 \cdot 5H_2O$	2.55	NDL
	Fe	100.00 $FeSO_4 \cdot H_2O$	32.86	NDL
	Mn	10.00 MnO	7.74	NDL
	Zn	140.00 $ZnSO_4 \cdot H_2O$	51.00	NDL
25	Cu	10.00 $CuSO_4 \cdot 5H_2O$	2.55	NDL
	Fe	100.00 $FeSO_4 \cdot H_2O$	32.86	NDL
	Mn	10.00 MnO	7.74	NDL
	Zn	140.00 $ZnSO_4 \cdot H_2O$	51.00	NDL
26	Cu	10.00 $CuSO_4 \cdot 5H_2O$	2.55	NDL
	Fe	100.00 $FeSO_4 \cdot H_2O$	32.86	NDL
	Mn	5.00 $MnSO_4$	1.82	NDL
	Zn	125.00 $ZnSO_4 \cdot H_2O$	45.53	NDL
27	Cu	10.00 $CuSO_4 \cdot 5H_2O$	2.55	NDL
	Fe	100.00 $FeSO_4 \cdot H_2O$	32.86	NDL
	Mn	5.00 $MnSO_4$	1.82	NDL
	Zn	125.00 $ZnSO_4 \cdot H_2O$	45.53	NDL
28	Cu	10.00 $CuSO_4 \cdot 5H_2O$	2.55	NDL
	Fe	NDL	–	NDL
	Mn	NDL	–	NDL
	Zn	115.00 ZnO	92.39	NDL
29	Cu	10.00 $CuSO_4 \cdot 5H_2O$	2.55	24.00
	Fe	NDL	–	150.00
	Mn	NDL	–	17.00
	Zn	100.00 ZnO	80.34	180.00
30	Cu	10.00 $CuSO_4 \cdot 5H_2O$	2.55	NDL
	Fe	NDL	–	NDL
	Mn	NDL	–	NDL
	Zn	100.00 ZnO	80.34	NDL

[1]NDL, not declared in the label.

Table S2. Declarations of macroelement content under "Analytical constituents" presented on the label (g/kg feed)[1].

Product	Amount declared under heading "Analytical constituents"				
	**Ca**	**P**	**K**	**Na**	**Mg**
1	12.00	9.00	NDL	NDL	NDL
2	11.62	11.30	NDL	NDL	NDL
3	22.30	10.50	NDL	NDL	NDL
4	10.00	5.00	NDL	NDL	NDL
5	24.10	15.00	NDL	NDL	NDL
6	15.00	10.00	4.90	3.90	1.50
7	12.00	10.00	NDL	NDL	NDL
8	14.60	13.40	3.80	3.50	NDL
9	8.00	NDL	NDL	NDL	NDL
10	15.00	10.00	8.50	3.50	1.60
11	13.00	10.00	NDL	NDL	NDL
12	15.00	10.00	NDL	NDL	NDL
13	12.50	9.00	NDL	NDL	NDL
14	16.00	9.00	NDL	NDL	NDL
15	8.00	7.00	NDL	NDL	0.90
16	11.00	8.00	NDL	4.50	NDL
17	14.50	9.50	NDL	4.00	NDL
18	16.00	10.50	NDL	3.50	NDL
19	15.00	11.00	7.00	3.00	1.30
20	15.00	11.00	7.00	3.20	1.10
21	15.00	11.00	NDL	NDL	NDL
22	21.00	12.00	NDL	NDL	NDL
23	21.00	11.00	NDL	NDL	NDL
24	15.00	10.00	NDL	NDL	NDL
25	14.00	9.00	NDL	NDL	NDL
26	20.00	12.00	NDL	NDL	NDL
27	20.00	13.00	NDL	NDL	NDL
28	10.50	8.50	5.00	3.00	1.10
29	15.00	11.00	8.50	4.50	1.00
30	14.60	13.40	3.80	3.50	NDL

[1]NDL, not declared in the label.
